# Facile synthesis of novel polypyrrole dispersed AgFeO_2_ nanohybrid with highly efficient photocatalytic activity towards 2,4,6-trichlorophenol degradation†

**DOI:** 10.1039/c8ra00754c

**Published:** 2018-04-10

**Authors:** Jyoti Kashyap, Ufana Riaz

**Affiliations:** Materials Research Laboratory, Department of Chemistry, Jamia Millia Islamia New Delhi-110025 India ufana2002@yahoo.co.in

## Abstract

With the aim to develop a visible light driven eco-friendly photocatalyst, the present work reports for the first time the synthesis of polypyrrole/AgFeO_2_ nanohybrids synthesized *via in situ* polymerization of pyrrole (by varying the mol ratios) in AgFeO_2_ dispersions. The nanohybrids were characterized using Fourier transform infrared (FT-IR) spectroscopy, ultra-violet visible near infrared (UV/VIS/NIR) spectroscopy, X-ray diffraction (XRD), thermogravimetric analysis (TGA) and transmission electron microscopy (TEM) measurements. The TGA measurements confirmed 20%, 60%, and 80% loading of pyrrole in AgFeO_2_ and hence the nanohybrids were designated as 20%-Ppy/AgFeO_2_, 60%-Ppy/AgFeO_2_, 80%-Ppy/AgFeO_2_ respectively. IR and X-ray photoelectron spectroscopy (XPS) studies confirmed polymerization of pyrrole and formation of the nanohybrids while XRD reflected high crystallinity of the nanohybrids. The photocatalytic activity of Ppy/AgFeO_2_ nanohybrids was investigated against 2,4,6-trichlorophenol (2,4,6-TCP) under sonophotocatalytic conditions using visible light irradiation. The nanohybrids were observed to completely degrade the organic pollutant within a short span of 40 min. The degradation kinetics fitted the pseudo-first order model. The fragments were analyzed using LCMS studies which revealed the formation of diols as degraded products. The nanohybrids revealed immense potential for rapid as well as eco-friendly destruction of organic pollutants in wastewater.

## Abstract 2

Synthesis of polypyrrole dispersed AgFeO_2_ nanohybrids for highly efficient visible light driven eco-friendly photocatalysis.

## Graphical abstract

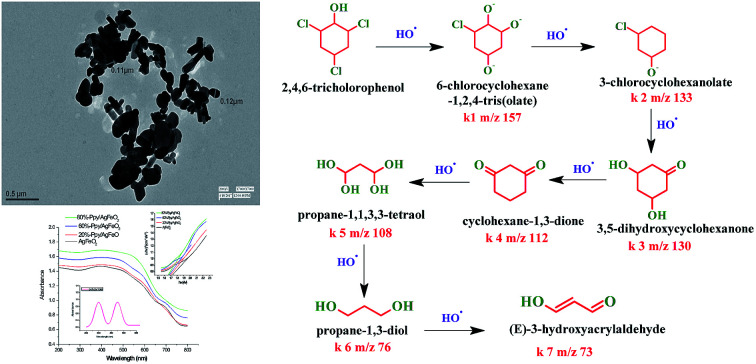

## Introduction

The rapid development of textile industry has significantly contributed to water pollution through the disposal of industrial wastes such as dyes and aromatic compounds into water bodies.^1,2^ 2,4,6-Trichlorophenol (TCP) is a well-known pollutant used by various pharmaceutical, paint, pesticide, and insecticide industries.^3,4^ Severe nervous system and respiratory disorders have been reported to be caused by the disposal of this organic pollutant in waste waters.^5,6^ Among the various methods adopted for water remediation, photocatalysis is proved to be one of the most eco-friendly and cost effective techniques applicable for rapid degradation of organic pollutants.^7,8^ Numerous semiconductor metal oxides/sulphides such as ZnO, TiO_2_ and CdS have been explored for their photocatalytic properties.^9,10^ Oxides of silver and gold are have also been investigated for their photocatalytic activity as they are non-toxic and possess inherent antimicrobial properties that could be used for treating water wasters for both dye as well as microbial degradation.^11,12^

Photocatalytic activity is mainly governed by recombination process and faster recombination of the photogenerated electron–hole pairs should be hindered for enhanced photocatalytic activity.^13^ Various techniques such as metallic doping, non-metallic doping and photosensitization through conducting polymers have been utilized to enhance the photocatalytic activity of the oxide semiconductors.^14–16^ Conducting polymer based organic–inorganic hybrid materials are promising photocatalysts due to their non-toxic nature, environmental stability, low cost, ease of preparation and excellent chemical stability.^16,17^

AgFeO_2_ (delafossite) is a non-toxic semiconductor oxide which has two kinds of polytypes, 3R and 2H structures.^18,19^ The band gap of AgFeO_2_ is reported to be in the range of 1.15–1.7 eV. However, the photocatalytic performance of this semiconductor is largely limited by its high electron–hole recombination rate.^20^ With the aim to improve the photocatalytic performance of this semiconductor *via* sensitization with a conducting polymer, the present work reports the synthesis of Ppy dispersed AgFeO_2_ nanohybrids through sonochemical polymerization. The nanohybrids were characterized using FTIR, UV-visible, XRD and TEM measurements. Sonophotocatalytic activity of PPy/AgFeO_2_ nanohybrids was explored by degrading 2,4,6-trichlorophenol under visible-light irradiation. The results demonstrated that the synthesized nanohybrids hold immense potential to be used as an effective photocatalyst under visible light irradiation.

## Experimental

Pyrrole (Sigma Aldrich, USA), 2,4,6-trichlorophenol (Himedia Chem. Pvt. Ltd., India), ferric nitrate (Loba Chemie, India), silver nitrate (Loba Chemie, India), ammonium hydroxide (Merck, India), ferric chloride (Sigma Aldrich, USA), methanol (Merck, India), ethanol (Merck, India), acetone (Merck, India) and *N*-methyl-2-pyrrolidone (NMP) (Merck, India) were used without further purification.

### Synthesis of polypyrrole (Ppy)

Pyrrole (0.050 mol) was added to ethanol (25 ml) in a 250 ml round bottom flask and stirred for 15 min at 30 °C. Ferric chloride (0.040 mol) dissolved in distilled water (25 ml) was then added to the above reaction mixture drop wise for a period of 30 min with continuous stirring at 30 °C. The reaction mixture was then sonicated for a period of 6 h at the same temperature. The suspension was then left undisturbed for 24 h at −5 °C overnight in a deep freezer. The polymer obtained was filtered, washed thoroughly with acetone and double distilled water to remove unreacted pyrrole and excess ferric chloride. A black precipitate of polypyrrole was obtained which was dried at 70 °C for 24 h in vacuum oven.

### Synthesis of silver ferrite (AgFeO_2_)

A mixture of silver nitrate (0.014 mol) and ferric nitrate (0.036 mol) were dissolved in deionized water (100 ml) and were stirred for 4 h at 27 °C on a magnetic stirrer. The hydroxide solution was then heated at 70 °C for 1 h. Ammonium hydroxide solution (50 ml) was slowly added to the above solution until a pH value of 12 was attained.^21^ The solutions were further stirred for 24 h and the resulting ruby-brown precipitate was collected by filtration. The precipitate was then washed several times with deionized water and dried at 60–70 °C in vacuum oven for 24 h. Ruby red color powder was obtained which was sintered at 700 °C in a muffle furnace to obtain the desired silver ferrite.

### Synthesis of Ppy/AgFeO_2_ nanohybrid

Nanohybrids of Ppy and AgFeO_2_ were prepared by homogeneously dispersing the synthesized silver ferrite (0.005 mol) in ethanol (50 ml) in a 250 ml round bottom flask. Pyrrole monomer (0.005 mol) was then added to the above solution followed by the addition of FeCl_3_·6H_2_O (0.004 mol) with continuous stirring at 30 °C. The reaction mixture was then sonicated for 8 h and the obtained nanohybrid was filtered, washed thoroughly with acetone and distilled water in order to remove the unreacted pyrrole and excess of ferric chloride. Similar procedure was adopted for the synthesis of nanohybrids of Ppy and AgFeO_2_ using AgFeO_2_ (0.005 mol), pyrrole (0.0025 mol) and (0.02 mol) respectively.

The amount of Ppy present in the nanohybrid ferrite was determined gravimetrically and compared with the weight loss obtained from thermogravimetric analysis (TGA). Ppy content was determined by the standard ash method as it was used as filler. The weight of each nanohybrid (*W*_0_) was measured using an analytical balance. The specimen was heated in an electric furnace at 1000 °C for 30 min to burn out the organic matrix and then re-weighed (*W*_1_). As the latter weight was only of the inorganic material, Ppy weight fraction (wt%) was determined according to the formula:Ppy loading = *W*_1_/*W*_0_ × 100%

The synthesized nanohybrids were therefore designated as 20%-Ppy/AgFeO_2_, 60%-Ppy/AgFeO_2_ and 80%-Ppy/AgFeO_2_ based on the weight measurements.

### Photocatalytic activity

Photocatalytic experiments were performed by decomposing 2,4,6-trichlorophenol (2,4,6-TCP) under visible light in a photochemical reactor (model LELESIL, India), fitted with an ultrasonic bath (model Soner 220H, 53 kHz, 500 W, M/S Scope Enterprises, India). A 300 W Xe arc lamp equipped with an ultraviolet cutoff filter was used to provide visible light with *λ* > 400 nm. The solutions were prepared in concentration of 75 ppm from a stock solution of 100 mg l^−1^. Prior to the exposure of visible irradiation, the suspension was stirred for a period 30 min to establish equilibrium and kept under dark conditions for about 1 h. A fixed amount of Ppy/AgFeO_2_ nanohybrid (150 mg) was added to TCP solution (200 ml) which was sonicated and exposed to visible light irradiation simultaneously for a period of 120 min. For the degradation analysis, aliquots (5 ml) of TCP solution were taken out at regular intervals (30 min, 60 min, 90 min and 120 min) and were centrifuged at a high speed (5000 rpm) using a centrifuge machine (model REMI R8C). Supernatant solutions were taken out and collected for determination of concentration of degraded dye which was monitored using a UV-visible spectrophotometer model Shimadzu UV-1800 at *λ*_max_ of 2,4,6-trichlorophenol (310 nm).

### Characterization

FT-IR spectra of the nanohybrids and the pristine compounds were taken on FT-IR spectrophotometer model Shimadzu IR Affinity-1 in the form of KBr pellets. UV-visible spectra were recorded on Lambda 750 S UV/VIS/NIR Spectrometer (Perkin Elmer, USA) using 2-propanol as solvent. X-Ray diffraction patterns were recorded on Philips PW 3710 powder diffractometer (Ni filtered Cu-Kα radiations). Peak parameters were analyzed using Origin 8.1 software. The *d* spacing was calculated using Bragg's equation. Transmission electron micrographs (TEM) were taken on Morgagni 268-D TEM, FEI, USA. The thermal stability of AgFeO_2_, Ppy and Ppy/AgFeO_2_ nanohybrid was investigated using thermal analyzer STA 6000, Perkin Elmer. The samples were heated from 30–800 °C at the rate of 10°C min^−1^ in N_2_ atmosphere at flow rate of 20 ml min^−1^. For the detection and identification of degradation products, liquid chromatography-mass spectroscopy (LC-MS) was conducted using a Waters XEVO G2-S TOF, USA mass spectrometer equipped with an electrospray ionization interface (ESI) source and operated in negative polarity mode fitted with BEH C18 (1.7 × 50 mm) containing 2.1 packed particles. ACN and mild Q waters containing 0.1% formic acid, pH 2.7, were used as eluents. The experiments were carried out in triplicate for evaluating the effect of nanohybrid catalyst dosage in the degradation of 2,4,6 trichlorophenol.

## Results and discussion

### Confirmation of loading of Ppy by TGA studies

The TGA profile of pure AgFeO_2_ revealed 1 wt% loss up to 840 °C showing its excellent thermal stability (given in ESI, Fig. S1†). The thermogram of pure Ppy revealed 50.2 wt% loss at 840 °C. The nanohybrid 20%-Ppy/AgFeO_2_ exhibited 10.5 wt% loss at 840 °C, while the nanohybrid 60%-Ppy/AgFeO_2_ revealed 32.2 wt% loss around the same temperature. Almost 38.6 wt% loss around 840 °C was noticed for the nanohybrid 80%-Ppy/AgFeO_2_. The TGA weight loss measurements for the nanohybrids were observed to be similar to the percentage of Ppy determined by ash method. The loading of Ppy in AgFeO_2_ was therefore confirmed. The nanohybrids revealed a highly good thermal stability even at 80% loading of Ppy.

### Confirmation of nanohybrid formation by IR analysis

The IR spectrum of AgFeO_2_ (given in ESI, Fig. S2(a)†) revealed a broad peak at 3434 cm^−1^ corresponding to the presence of OH groups on the iron oxide surface.^10^ The peak at 1629 cm^−1^ was attributed bending vibrations of O–H for group bound to Fe atoms while peaks at 1062 cm^−1^ and 684 cm^−1^ corresponded to Fe–O vibration modes.^21^ Two strong peaks were observed at 550 cm^−1^ and 475 cm^−1^ that were assigned to the stretching vibrations of the Ag–O bonds and the Fe–O. bonds, respectively.^21^ The IR spectrum of Ppy, revealed N–H stretching vibration peak centered at 3415 cm^−1^, C–C asymmetric stretching at 1450 cm^−1^, and pyrrole ring stretching at 1538 cm^−1^ (given in ESI, Fig. S2(b)†).^21,22^ The peak at 1039 cm^−1^ was attributed to C–H in-plane deformation, the peak at 1164 cm^−1^ was correlated to N–C stretching vibration, while the peak at 1304 cm^−1^ was associated with C–H in-plane vibration. The peak at 1164 cm^−1^ was attributed to C–N stretching vibration, and the peak at 1175 cm^−1^ revealed the in-plane deformation vibration of NH^+^, which is formed on the polymer chains by protonation. The peaks at 1039 cm^−1^ and 901 cm^−1^ were observed due to the presence of C–H and N–H in-plane deformation vibrations and C–C out-of-plane ring deformation vibration respectively. The peaks correlated to ring deformation were noticed at 906 cm^−1^, 895 cm^−1^, 781 cm^−1^ and 674 cm^−1^. The IR spectrum of the nanohybrid 20%-Ppy/AgFeO_2_, (ESI, Fig. S2(b)†) showed shifting of the peaks corresponding to Ppy and AgFeO_2_. A broad NH stretching peak was noticed around 3424 cm^−1^ due to interaction between the –NH group of Ppy with Fe^2+^ ions of the AgFeO_2_ suggesting that each AgFeO_2_ particle was completely coated with Ppy. The C–C asymmetric stretching peak was noticed at 1401 cm^−1^, while pyrrole ring stretching peaks were noticed at 1558 cm^−1^, 1216 cm^−1^ and 1048 cm^−1^ respectively. Strong absorption peaks were noticed at 537 cm^−1^ and 460 cm^−1^ which were assigned to the stretching vibrations of the Fe–O bonds in tetrahedral positions and the Ag–O bonds, respectively.^19,20^ However, upon increasing the loading of Ppy upto 60%, shifting of N–H vibration peak to 3434 cm^−1^ was noticed while the characteristic peaks attributed to AgFeO_2_ were observed at 537 cm^−1^, and 460 cm^−1^. The nanohybrid 80%-Ppy/AgFeO_2_ exhibited a pronounced shift in the N–H stretching vibration peak which was observed at 3454 cm^−1^ indicating strong interaction of the Fe–O and Ag–O bond of AgFeO_2_ with Ppy. The presence of the peaks associated with polypyrrole as well as AgFeO_2_ confirmed polymerization of the former as well as formation of the nanohybrid.^21^

### Analysis of electronic transitions of Ppy/AgFeO_2_*via* UV-visible studies

**Fig. 1:**
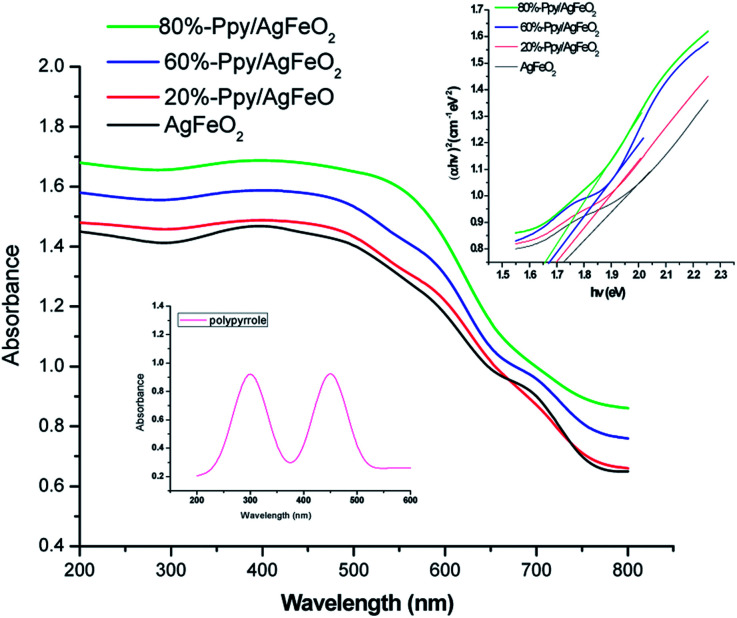
UV-visible spectra of polypyrrole (inset) and Ppy/AgFeO_2_ nanohybrids (Tauc plot in inset).

The UV-vis spectrum of Ppy, Fig. 1(inset) showed two distinct absorption peaks; one at 300 nm correlated to the π–π* transition of aromatic C=C bonds and other absorption peak at about 450 nm associated with polaronic transitions.^22^ The UV spectrum of AgFeO_2_, Fig. 1, revealed a broad adsorption region in ultraviolet and visible light and the band gap (*E*_g_) was calculated to be 1.73 eV as reported by other authors (shown in inset).^23^ The UV-visible spectra of the Ppy/AgFeO_2_ nanohybrids revealed an increase in the intensity of absorption band corresponding to AgFeO_2_ with the increase in the loading of Ppy. The intensity of this peak was noticed to be highest for 80%-Ppy/AgFeO_2_ suggesting intense interaction of the two components with the increase in the loading of Ppy, while it was observed to be lowest for 20%-Ppy/AgFeO_2._. The band gap was calculated to be 1.65 eV, 1.68 eV and 1.7 eV for 80%-Ppy/AgFeO_2_, 60%-Ppy/AgFeO_2_ and 20%-Ppy/AgFeO_2_ respectively, confirming synergistic interaction between Ppy and AgFeO_2_ that improved the optical properties of the nanohybrid.

### Morphological analysis of Ppy/AgFeO_2_ nanohybrids *via* TEM and XRD

The TEM of Ppy (given in ESI, Fig. S3(a)†), revealed the formation of a dense fused cluster while the TEM of AgFeO_2,_ exhibited a fused tubular morphology with particles ranging between 90–100 nm (ESI Fig. S3(b)†). The TEM of 20%-Ppy/AgFeO_2_, (ESI Fig. S3(c)†) showed the predominance of AgFeO_2_ morphology revealing the formation of tubular structures of dense as well as hollow particles ranging between 50–60 nm. The TEM of 60%-Ppy/AgFeO_2_ (ESI Fig. S3(d)†) also showed a tubular morphology but the size of the particles was found to be bigger than the previous nanohybrid ranging 100–150 nm. The morphology of 80%-Ppy/AgFeO_2_, (ESI Fig. S3(e)†) exhibited the formation of distorted dense as well as hollow spheres joining together to from a huge cluster. It can therefore be concluded that at lower loading of Ppy, the morphology of AgFeO_2_ was found to be predominant while at higher loadings, the morphology revealed aggregation and formation of clusters of Ppy as well as AgFeO_2_ particles.

**Fig. 2:**
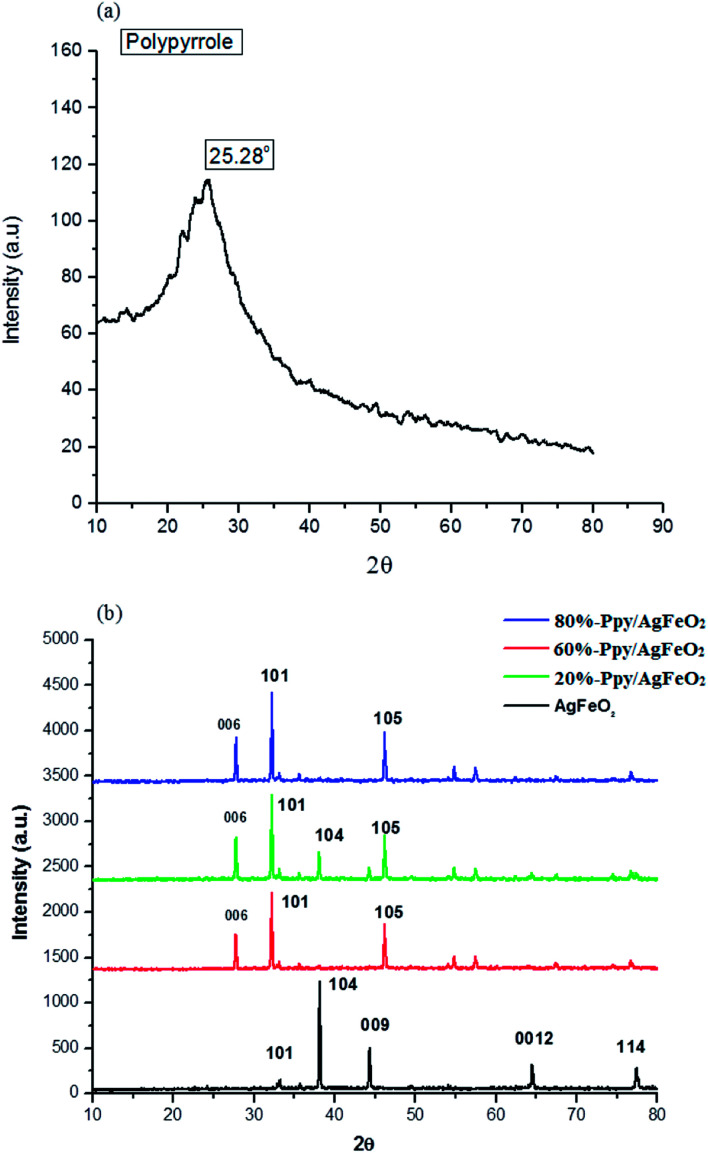
XRD profiles of (a) Ppy (b) AgFeO_2_,Ppy/AgFeO_2_ nanohybrids.

The XRD of profile of pure Ppy, Fig. 2(b), revealed a broad peak at 2*θ* = 25.28°, confirming the amorphous nature of the polymer.^24^ The XRD profile of AgFeO_2_, Fig. 2(a), showed peaks at 2*θ* = 32.36°, 38.26°, 44.30°, 64.57°, 77.40°assigned to scattering from *d*(101), *d*(104), *d*(009), *d*(0012), *d*(114), planes, respectively. The (104) reflections were intense suggesting the preferred orientation of the grains along the *c*-axis. All diffraction peaks were correlated to the presence of α-AgFeO_2_ which is predominantly a 3R structure (JCPDS: 75-2147) though a small amount of 2H structure was also present (JCPDS: 25-0765).^18,19^ The peaks associated with metallic Ag, Ag_2_O, and FeOOH were not found which confirmed the formation of delafossite type structure. The XRD pattern, of Ppy/AgFeO_2_ nanohybrids, Fig. 2(b), showed the appearance of a new peak at 2*θ* = 27.76° corresponding to *d*(006) plane. This peak appeared to be quite pronounced in the nanohybrids, showing the dominance of 2H type polystructure of AgFeO_2_. The intensity of the peak observed at 2*θ* = 32.36° corresponding to *d*(101) plane also appeared to be quite prominent in case of the nanohybrids besides the other peaks.

The appearance of the *d*(006) plane and the increase in the intensity of the peak corresponding to *d*(101) plane were attributed to the changes in the lattice symmetry of the AgFeO_2_, as the 3R polytype has rhombohedral symmetry, while the 2H polytype consists of stacking alternate layers with hexagonal symmetry. The 2H delafossite possesses a layered structure with one layer composed of close-packed Ag^+^ ions and the other as edge-shared Fe^3+^O_6_ octahedra containing the iron cations inside. The Ag^+^ ions are coordinated by two oxygen ions with the linear O–Ag–O bonds while, each of the Fe^3+^ cation is coordinated with six oxygen ions. It can therefore be concluded that the dispersion of Ppy in AgFeO_2_ was found to change the symmetry of the later, while the overall morphology of the nanohybrids was observed to be crystalline.

### XPS analysis

**Fig. 3:**
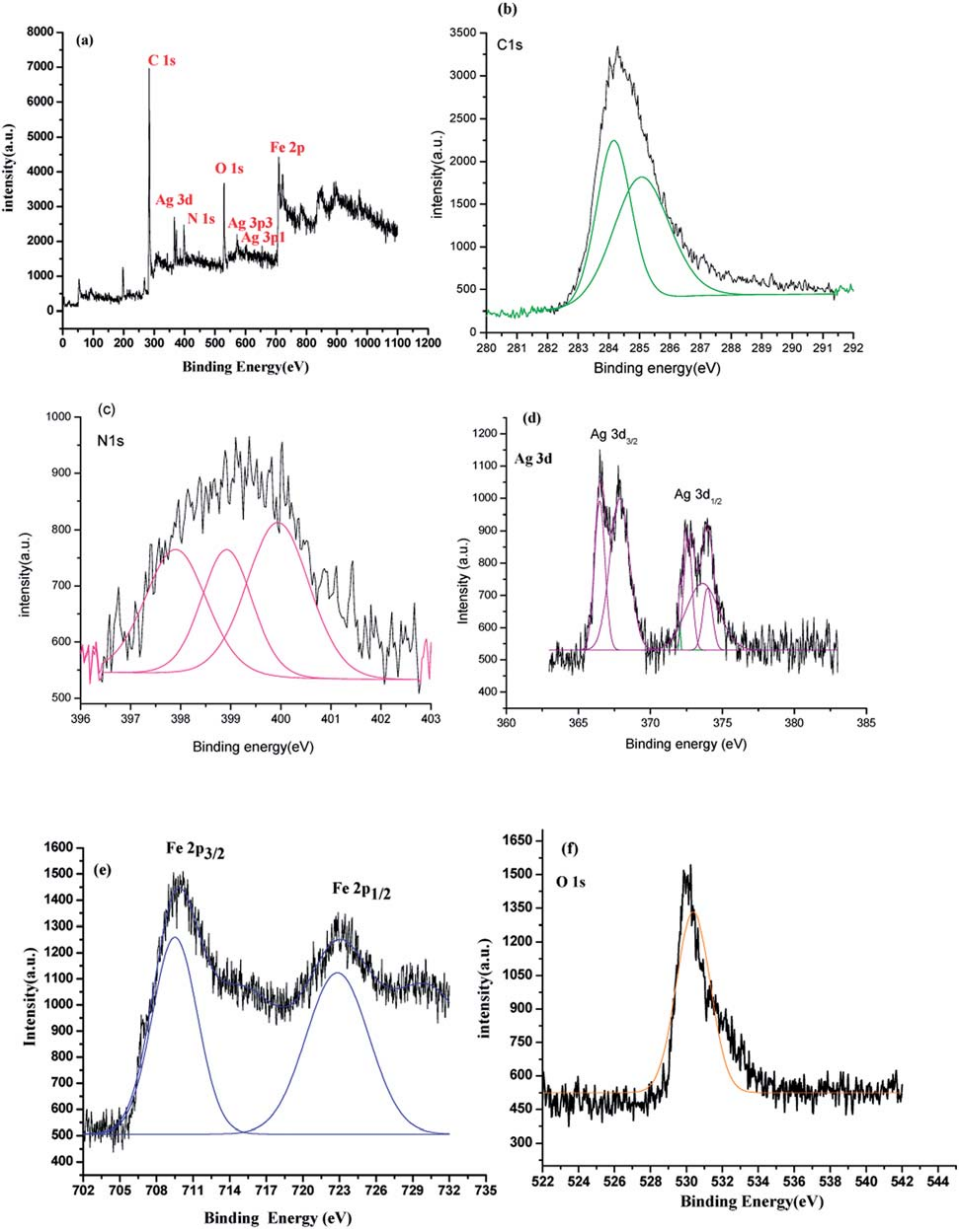
XPS survey of (a) 80%-Ppy/AgFeO_2_ nanohybrid and high resolution XPS of (b) C 1s (c) N 1s (d) Ag 3d (e)Fe 2p (f) O 1s.

X-ray photon spectroscopy (XPS) is an effectual tool for analyzing elements and their corresponding valence state. The XPS of 80%-Ppy/AgFeO_2_,Fig. 3(a), revealed the presence of elements of Ag 3d, Fe 2p, O 1s, C 1s and N 1s. The C 1s and N 1s peaks were correlated to the presence Ppy, Fig. 3(b) and (c). The C 1s peaks were observed at 284.1 eV and 285.2 eV respectively. The former peak was correlated to the β-carbons of the Py ring, while the later peak was associated with the presence of α-carbon. The N 1s peak, Fig. 3(c), appeared as triplet with binding energies 397.5 eV, 399.5 eV and 401.3 eV respectively, which indicated the presence of sp^2^-bonded nitrogen (C–N=C), neutral nitrogen atoms (NH) and positively charged nitrogen atoms (N^+^) in Ppy further confirming the co-existence of Ppy in the nanohybrid.^23^ The Ag 3d_3/2_ and Ag 3d_5/2_ showed split doublet peaks at 367.8 eV, 368.5 eV, 373.5 eV and 374.8 eV respectively, Fig. 3(d). The peaks centered at 367.8 and 373.5 eV were attributed to the presence of Ag^+^ in AgFeO_2_, while the peaks at 368.5 eV and 374.8 eV were correlated to the presence of Ag^0^ on the surface of AgFeO_2_. These binding energies values were found to be in good agreement with the reported values.^23^ The XPS spectrum Fe 2p showed doublets at 710.5 eV (2p_3/2_) and 723.9 (2p_1/2_), Fig. 3(e), demonstrating the existence of Fe^3+^ ions.^23^ The peak at 531.5 eV observed in the XPS spectrum of O 1s, Fig. 3(f), was attributed to the presence the lattice oxygen in AgFeO_2_.^23^ The presence of the peaks associated with Ppy as well as AgFeO_2_ confirmed the formation of nanohybrid.

### Evaluation of sonophotocatalytic properties of Ppy, AgFeO_2_ and Ppy/AgFeO_2_ nanohybrids

**Fig. 4:**
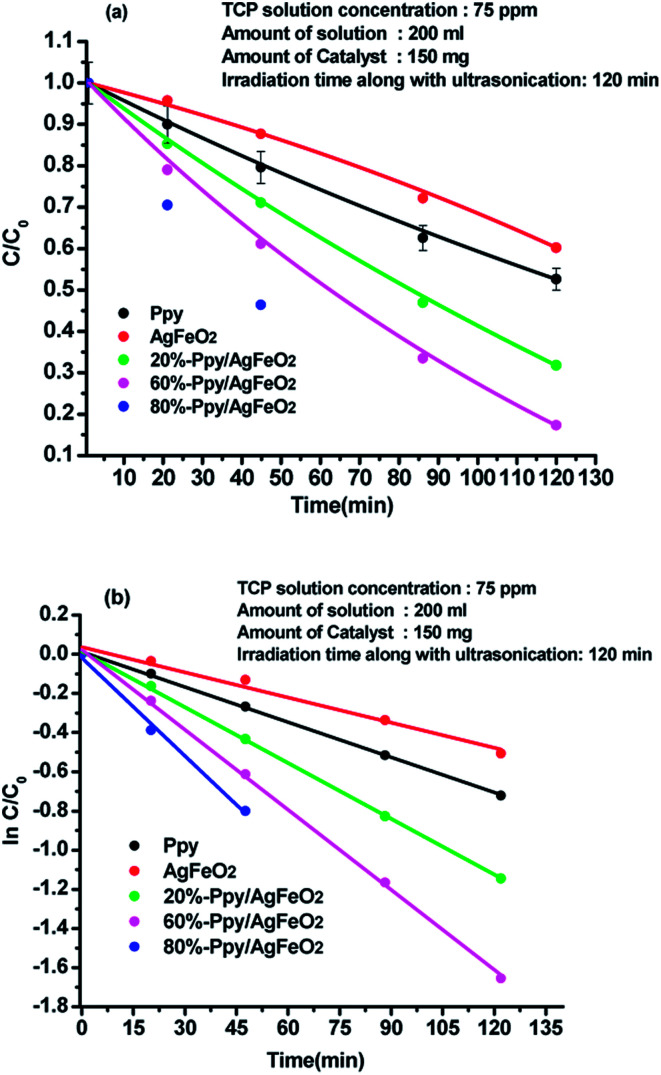
Plot of (a) *C*/*C*_o_ and (b) ln *C*/*C*_o_*versus* time in acidic medium for 2,4,6-TCP solution (75 ppm) using Ppy, AgFeO_2_ and Ppy/AgFeO_2_ nanohybrids.

Degradation of 2,4,6-trichlorophenol (TCP) was performed by sonophotocatalysis and was monitored by UV-vis spectroscopy. The peaks associated with 2,4,6-trichlorophenol were observed at 242 nm and 310 nm, (ESI Fig. S4(a)–(e)†). In presence of pure Ppy, 47.33% degradation was achieved while in presence of AgFeO_2_, 45.83 wt% degradation was recorded. Around 68.14% degradation was attained using 10%-Ppy/AgFeO_2_ after 140 min under visible light irradiation. Maximum degradation of TCP was found to be 99.6% in 120 min in the presence of 80%-Ppy/AgFeO_2_, while in presence of 30%-Ppy/AgFeO_2_, it was observed to be 82.23%. It can be seen that the photocatalytic efficiency of Ppy/AgFeO_2_ nanohybrid was higher than that of neat Ppy and AgFeO_2_. With the increase in the loading of Ppy in AgFeO_2_, the photocatalytic efficiency increased. The degradation kinetics data adequately fitted the pseudo-first order model in Ppy, AgFeO_2_, Ppy/AgFeO_2_ nanohybrids, with high correlation coefficients values of 0.998 and 0.981, 0.999, 0.999, 0.990 respectively, Fig. 4(a) and (b). The rate constant was calculated to be 0.005 min^−l^ and 0.004 min^−l^ for pure Ppy and AgFeO_2_ respectively. Incase of the nanohybrids the rate constant was observed to be highest for 80%-Ppy/AgFeO_2_ (*k* = 0.016 min^−1^).

### Effect of catalyst concentration on TCP degradation

**Fig. 5:**
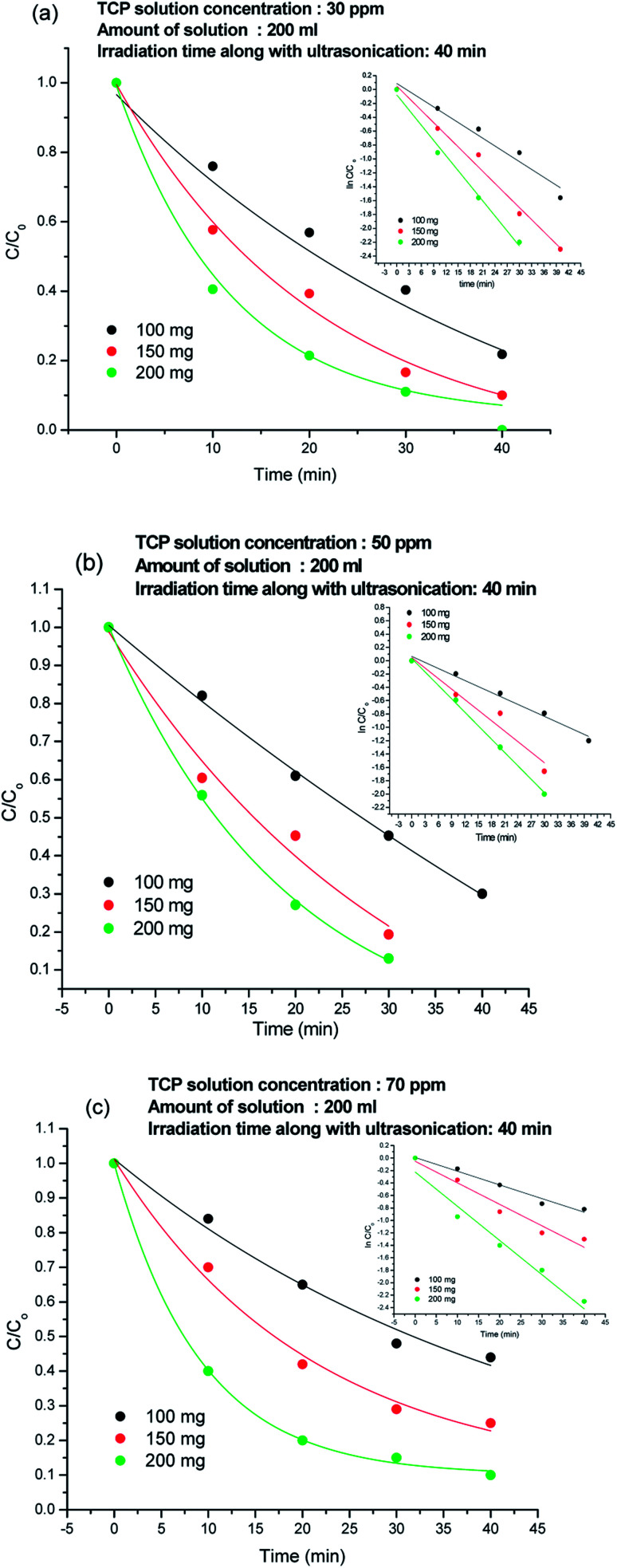
Degradation plots of TCP solution using 100 mg, 150 mg and 200 mg 80%-Ppy/AgFeO_2_ nanohybrid in (a) 30 ppm solution, (b) 50 ppm solution and (c) 70 ppm solution.

The *C*/*C*_o_ plots of the TCP degradation were studied by taking TCP solutions of 30 ppm, 50 ppm, 70 ppm concentrations in presence of 100 mg, 150 mg, 200 mg 80%-Ppy/AgFeO_2_ nanohybrid catalyst for a period of 40 min as shown in Fig. 5(a)–(c). For 30 ppm TCP solution, Fig. 5(a), the nanohybrid revealed 84% degradation in 40 min using 100 mg catalyst, while 95% degradation was achieved using 150 mg and 200 mg catalyst within a period of 40 min. Likewise 50 ppm and 70 ppm TCP solutions showed almost 94% and 90% degradation respectively, when 200 mg of the nanohybrid catalyst was used, Fig. 5(b) and (c). The degradation efficiency was found to be high even at lower loading of the catalyst. The rate constant (*k*) values using 100 mg, 150 mg, and 200 mg of the nanohybrid was calculated to be 0.037 min^−1^, 0.038 min^−1^, 0.072 min^−1^ respectively for 30 ppm TCP solution, 0.029 min^−1^, 0.056 min^−1^, and 0.067 min^−1^ for 50 ppm TCP solution and 0.013 min^−1^, 0.018 min^−1^, 0.025 min^−1^ respectively for 70 ppm TCP solution. The degradation was observed to follow pseudo first order kinetics in all cases.

### Effect of pH of TCP solution on the degradation efficiency

**Fig. 6:**
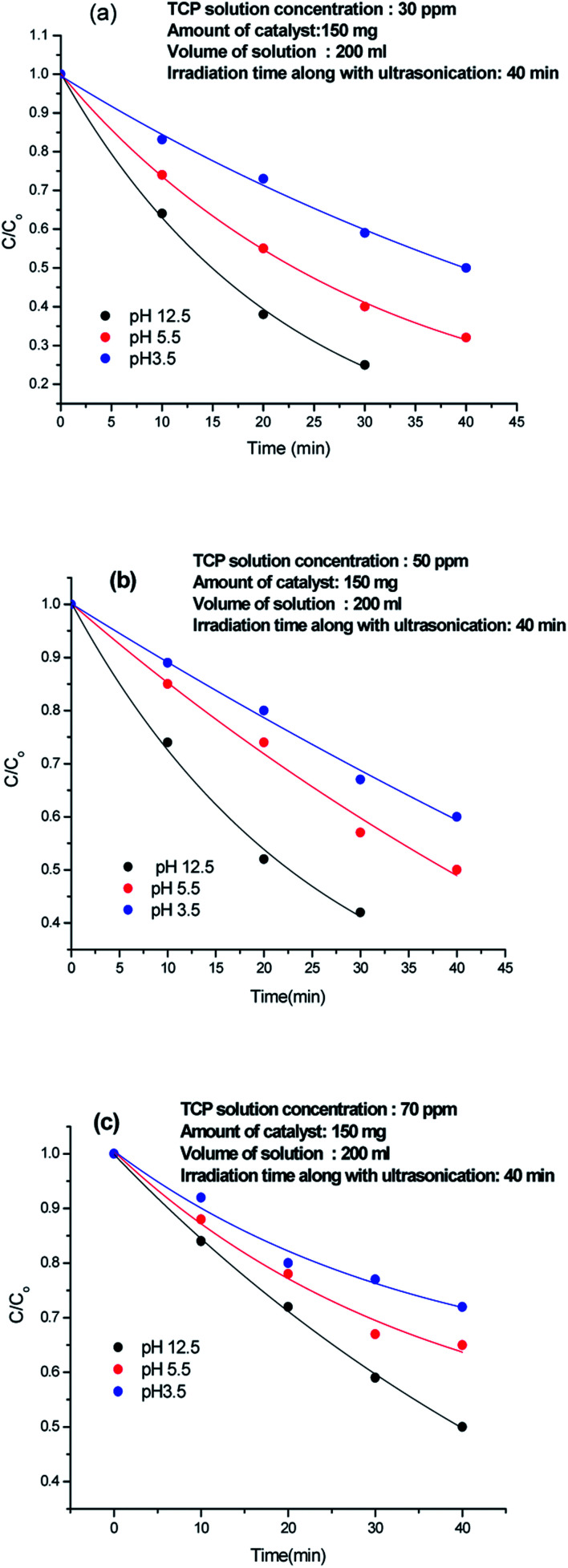
Effect of pH of TCP solution on the degradation efficiency.

The effect of pH on the degradation was analysed by taking 30 ppm, 50 ppm, and 70 ppm TCP solutions of pH 3.5, 5.5 and 12.5 respectively, as shown in Fig. 6(a)–(c). At 3.5, 50% degradation occurred incase of 30 ppm TCP solution, Fig. 6(a), while 40% and 30% degradation was achieved for 50 ppm and 70 ppm solutions respectively,Fig. 6(b) and (c). At pH 5.5, 70% degradation was observed for 30 ppm TCP solution, whereas 50 ppm and 70 ppm TCP solution revealed 50% and 35% degradation respectively. Around 75% degradation was noticed for 30 ppm TCP solution at pH 12.5, Fig. 6(a), while 60% and 50% degradation efficiencies were observed for 50 ppm and 70 ppm TCP solutions taken at the same pH. The degradation efficiency was observed to be higher in basic medium as compared to acidic medium. The dissociation constant (p*K*_a_) value for TCP is reported to be 6 and therefore at pH < 6, the molecule will exist in its undissociated form while at pH > 6, it will exist as trichlorophenolate ion. Hence, the increase in the degradation efficiency in alkaline medium can be attributed to the presence of presence of excess hydroxyl ions which can form hydroxyl radicals (HO˙) by trapping the photo electrons produced during visible light illumination. These hydroxyl radicals can easily degrade the trichlorophenolate anions as compared to the undissociated TCP molecule.^25^

**Fig. 7:**
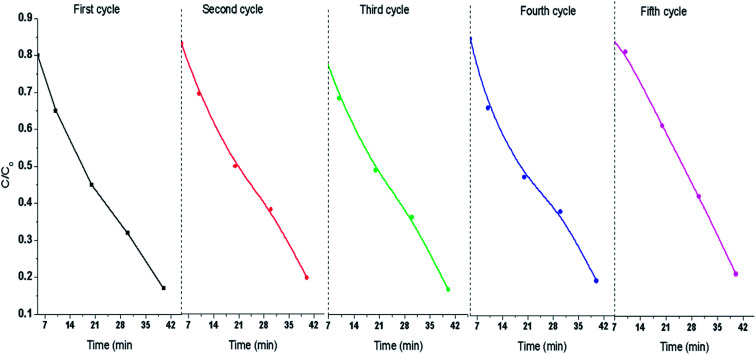
Recyclability of 80%-Ppy/AgFeO_2_ catalyst.

The recyclability of 80%-Ppy/AgFeO_2_ was tested upto five cycles and each cycle was carried out for a period of 40 min, Fig. 7. It was observed that 80%-Ppy/AgFeO_2_ nanohybrid revealed 85% degradation upto four cycles while 80% degradation was noticed after fourth cycle. The nanohybrid was found to exhibit efficient reusability by maintaining its photocatalytic activity up to four recycles under visible light illumination.

### Proposed degradation pathway of 2,4,6-trichlorophenol

**Scheme 1:**
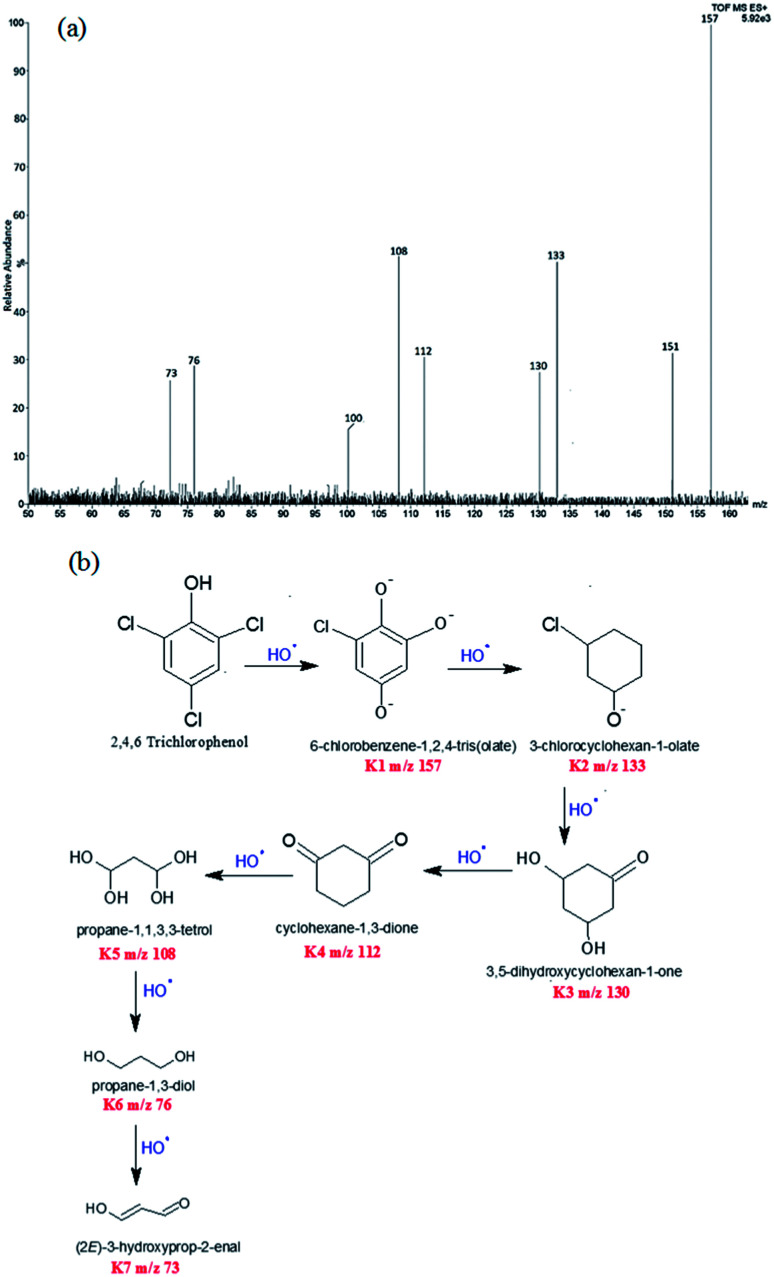
(a) LCMS spectrum of intermediates of 2,4,6-trichlorophenol (b) degradation pathway of 2,4,6-trichlorophenol.

Degradation of TCP was confirmed by LCMS studies which revealed a variety of intermediate compounds formed during the course of the reaction, Scheme 1(a). The intermediates with their increasing *m*/*z* values are shown in Scheme 1(b). First intermediate was assigned 100% abundance. Intermediates of 100% abundance with low *m*/*z* values ranging from 160–70 were obtained. They were labelled as K1, K2, K3, K4, K5, K6, and K7. The first intermediate K1 (*m*/*z* 157) showed 100% abundance and was taken as the main degradation product.

Intermediates with *m*/*z* values 151 (35%), 133 (50%), 130 (28%), 112 (33%), 108 (54%), 100 (19%), 76 (31%) and 73 (29%) were obtained. These intermediates revealed that degradation proceeded *via* substitution of Cl^−^ with O^−^, through the attack of HO˙ free radicals. Interestingly the HO˙ radicals preferentially attacked the electron rich chloride functionality of the TCP molecule to form 6-chlorobenzene-1,2,4-tris(olate) (K1, *m*/*z* 157). This fragment then degraded into 3-chlorocyclohexan-1-olate (K2, *m*/*z* 133). 3,5-Dihydroxycyclohexan-1-one (K3, *m*/*z* 130), was obtained from K2 fragment which was found to undergo oxidation to form cyclohexane-1,3-dione (K4, *m*/*z* 112). The degradation product K5 was obtained by ring opening of cyclohexane *via* the attack of ˙OH free radicals, Scheme 1(b). Lowest fragment obtained was propane-1,3-diol (K7, *m*/*z* 73).

## Conclusion

Polypyrrole/AgFeO_2_ nanohybrids were successfully synthesized *via in situ* chemical polymerization method. IR and UV studies confirmed the formation of the nanohybrid while XRD studies revealed the presence of mixed 2H and 3R polytype structures of AgFeO_2_ upon nanohybrid formation. TEM showed intense cluster formation upon higher loading of Ppy in AgFeO_2_. The photocatalytic activity of the nanohybrid was explored for the degradation of 2,4,6-trichlorophenol. The nanohybrid was found to exhibit high photocatalytic activity under visible light illumination and TCP was found to degrade within a short span of 40 min. The analysis of the degraded fragments revealed that the obtained products were diols which are potentially non-toxic. Hence, the synthesized hybrids can be safely used for the eco-friendly degradation of toxic organic pollutants.

## Conflicts of interest

There are no conflicts to declare.

## Supplementary Material

RA-008-C8RA00754C-s001

## References

[cit1] Riaz U., Ashraf S. M., Kashyap J. (2015). Mater. Res. Bull..

[cit2] Riaz U., Ashraf S. M., Kashyap J. (2015). Polym.-Plast. Technol. Eng..

[cit3] Shirgaonkar Z., Pandit A. (1998). Ultrason. Sonochem..

[cit4] Tan I. A. W., Ahmad A. L., Hameed B. H. (2009). J. Hazard. Mater..

[cit5] Rengaraj S., Li X. Z. (2006). J. Mol. Catal. A: Chem..

[cit6] Pandit A. B., Gogate P. R., Majumdar S. (2001). Ultrason. Sonochem..

[cit7] Hoffmann M. R., Martin S. T., Choi W. Y., Bahnemannt D. W. (1995). Chem. Rev..

[cit8] Pekakis P. A., Xekoukoulotakis N. P., Mantzavinos D. (2006). Water Res..

[cit9] Riaz U., Ashraf S. M., Budhiraja V., Aleem S., Kashyap J. (2016). J. Mol. Liq..

[cit10] Riaz U., Ashraf S. M. (2012). Sep. Purif. Technol..

[cit11] Rosseeler O., Shankar M. V., Du M. K. L., Schmidlin L., Keller N., Keller V. (2010). J. Catal..

[cit12] Chaudhary D., Khare N., Vankar V. D. (2016). Ceram. Int..

[cit13] Riaz U., Ashraf S. M. (2015). RSC Adv..

[cit14] Zheng Z., Huang B., Meng X., Wang J., Wang S., Lou Z., Wang Z., Qin X., Zhang X., Dai Y. (2013). Chem. Commun..

[cit15] Zhang Q., Lima D. Q., Lee I., Zaera F., Chi M., Yin Y. (2011). Angew. Chem., Int. Ed..

[cit16] Riaz U., Ashraf S. M., Aqib M. (2014). Arabian J. Chem..

[cit17] Riaz U., Ashraf S. M. (2014). RSC Adv..

[cit18] Ong K. P., Bai K. W., Blaha P., Wu P. (2007). Chem. Mater..

[cit19] Ong K. P., Bai K. W., Wu P. (2008). J. Alloys Compd..

[cit20] Tang D. D., Zhang G. K. (2017). Appl. Surf. Sci..

[cit21] Riaz U., Ashraf S. M., Raza R., Kohli K., Kashyap J. (2016). Ind. Eng. Chem. Res..

[cit22] Chaudhary M., Islam R., Witcomb M. J., Mallick K. (2014). Dalton Trans..

[cit23] Tang D., Zhang G. (2017). Ultrason. Sonochem..

[cit24] Wang J.-G., Wei B., Kang F. (2014). RSC Adv..

[cit25] Tanaka S., Saha U. K. (1994). Water Sci. Technol..

