# Supplementary material for: Facile synthesis of novel polypyrrole dispersed AgFeO2 nanohybrid with highly efficient photocatalytic activity towards 2,4,6-trichlorophenol degradation
Source: RSC Adv. 2018 Apr 10;8(24):13218–25. doi: 10.1039/c8ra00754c (PMC9079723; doi:10.1039/c8ra00754c)
Supplement: RA-008-C8RA00754C-s001 [file RA-008-C8RA00754C-s001.pdf]

Electronic Supplementary Material (ESI) for RSC Advances.
This journal is © The Royal Society of Chemistry 2018

Supporting Information

Facile Synthesis of Novel Polypyrrole dispersed $AgFeO_2$ Nanohybrid with Highly Efficient Photocatalytic Activity towards 2,4,6-Trichlorophenol Degradation

Jyoti Kashyap, and Ufana Riaz*

Materials Research Laboratory, Department of Chemistry, Jamia Millia Islamia , New Delhi-110025,India,Corresponding author email : ufana2002@yahoo.co.in

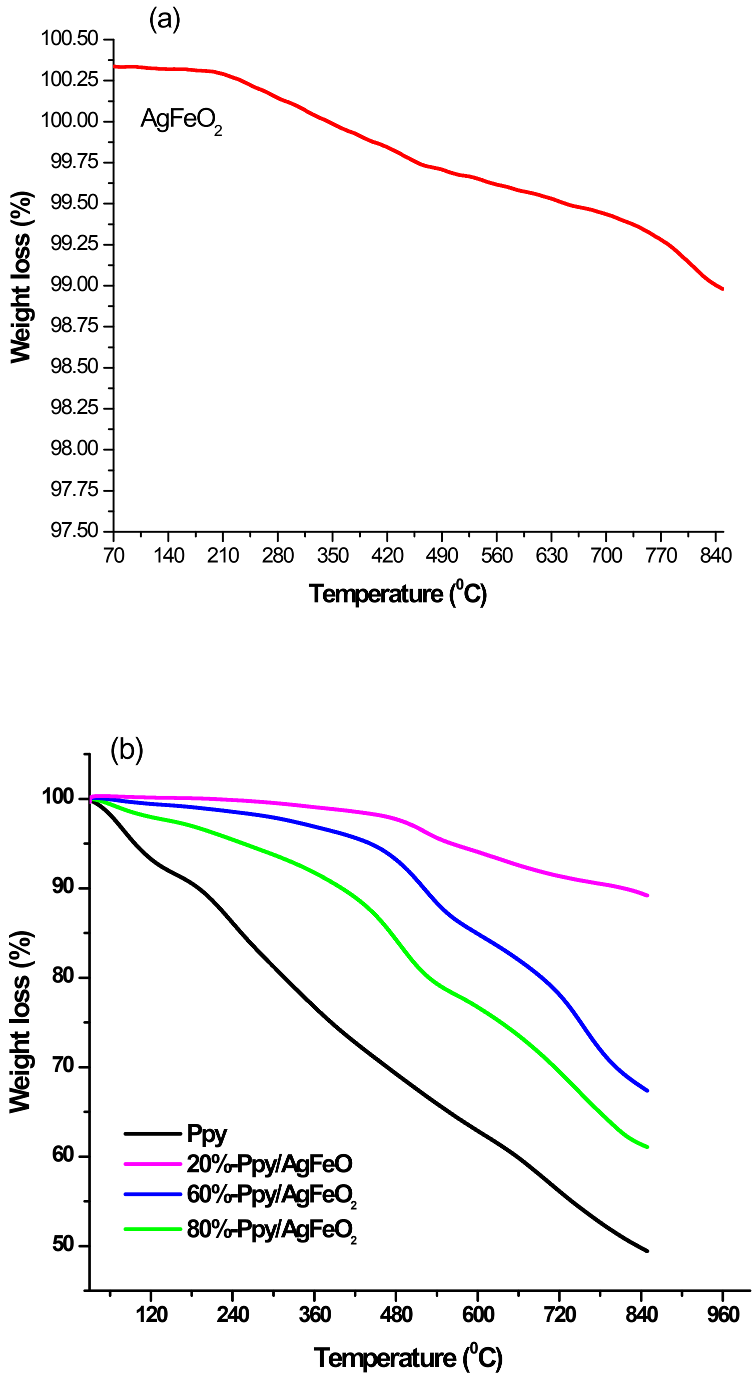

Figure S1 TGA profiles of (a) $AgFeO_2$ (b) Ppy, and Ppy/$AgFeO_2$ nanohybrids

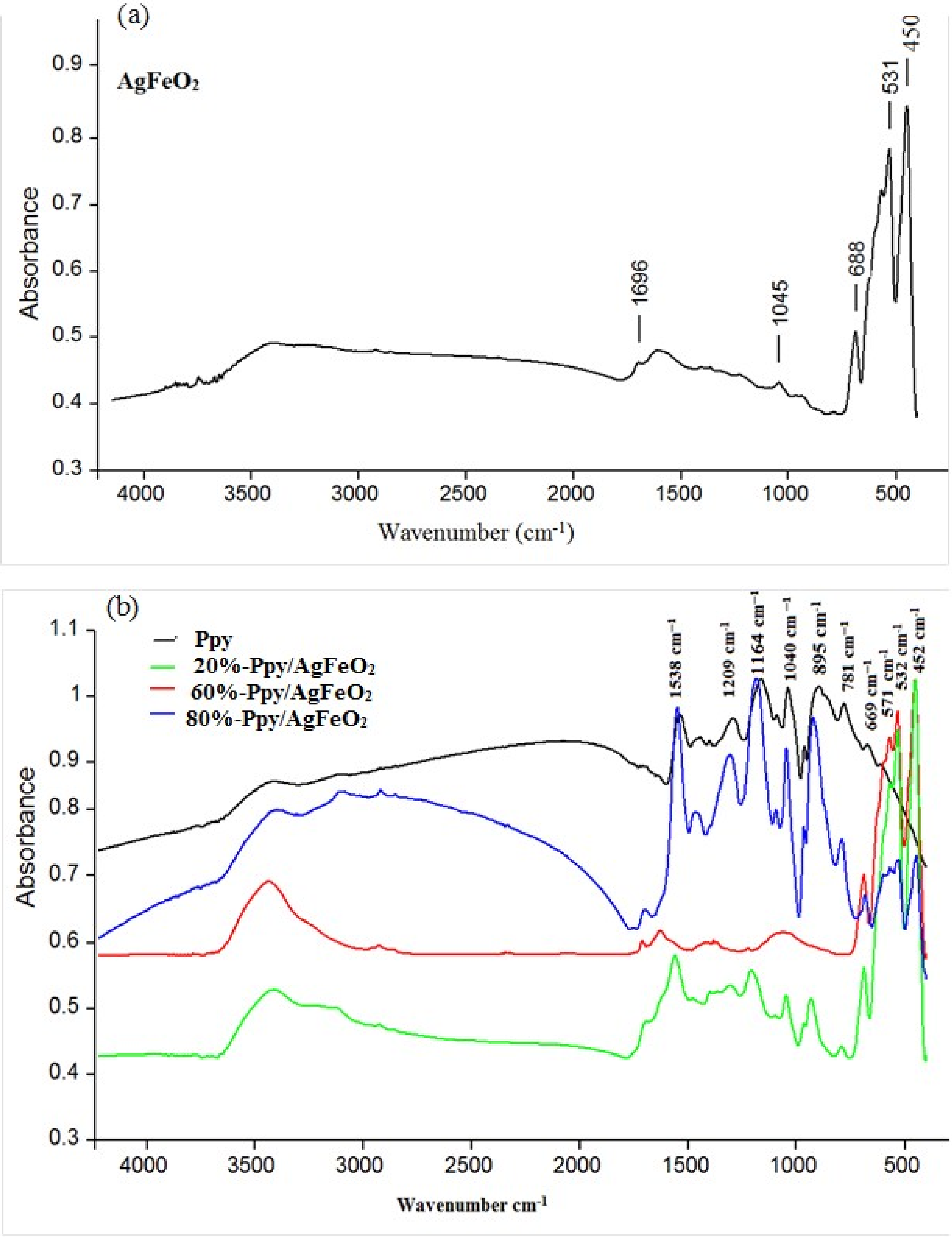

Figure S2 IR spectra of (a) $AgFeO_2$ (b) Ppy/$AgFeO_2$ nanohybrids

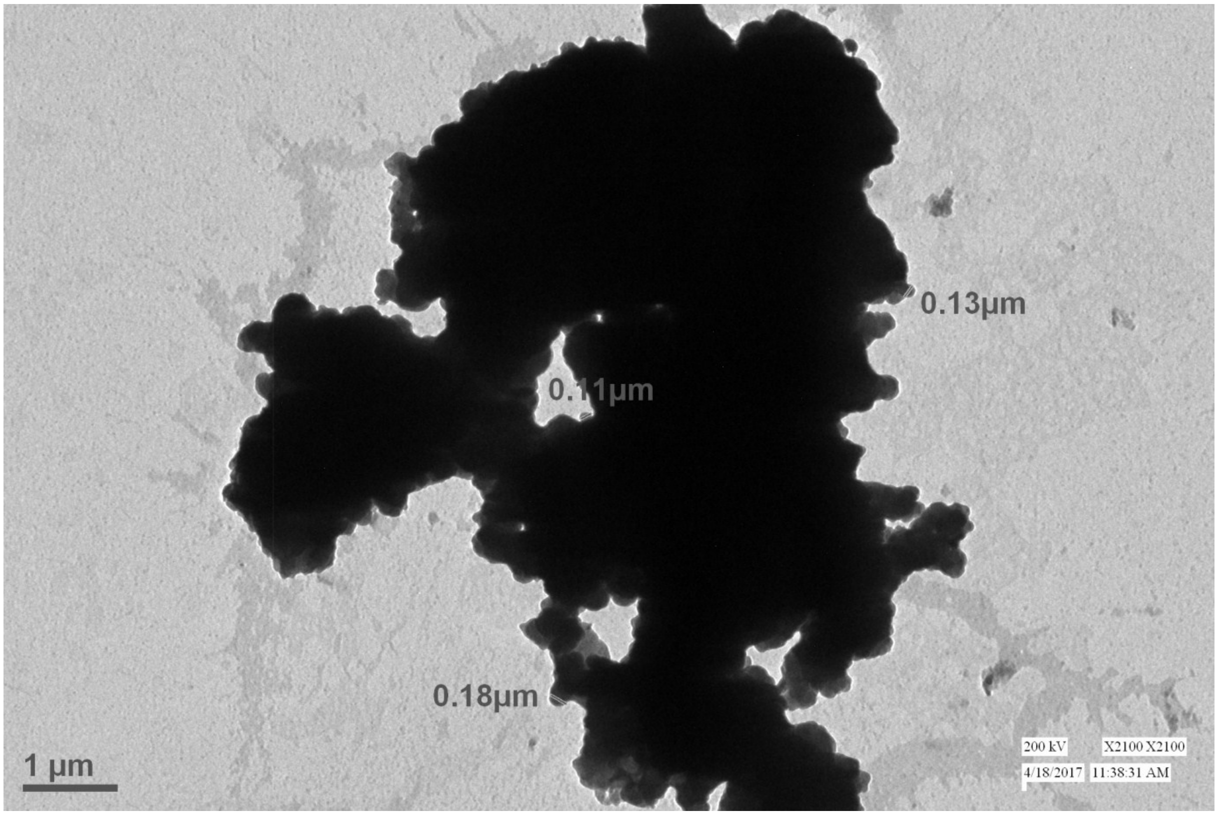
0.13μm
0.11μm
0.18μm
1 μm
200 kV
X2100 X2100
4/18/2017
11:38:31 AM

(a)

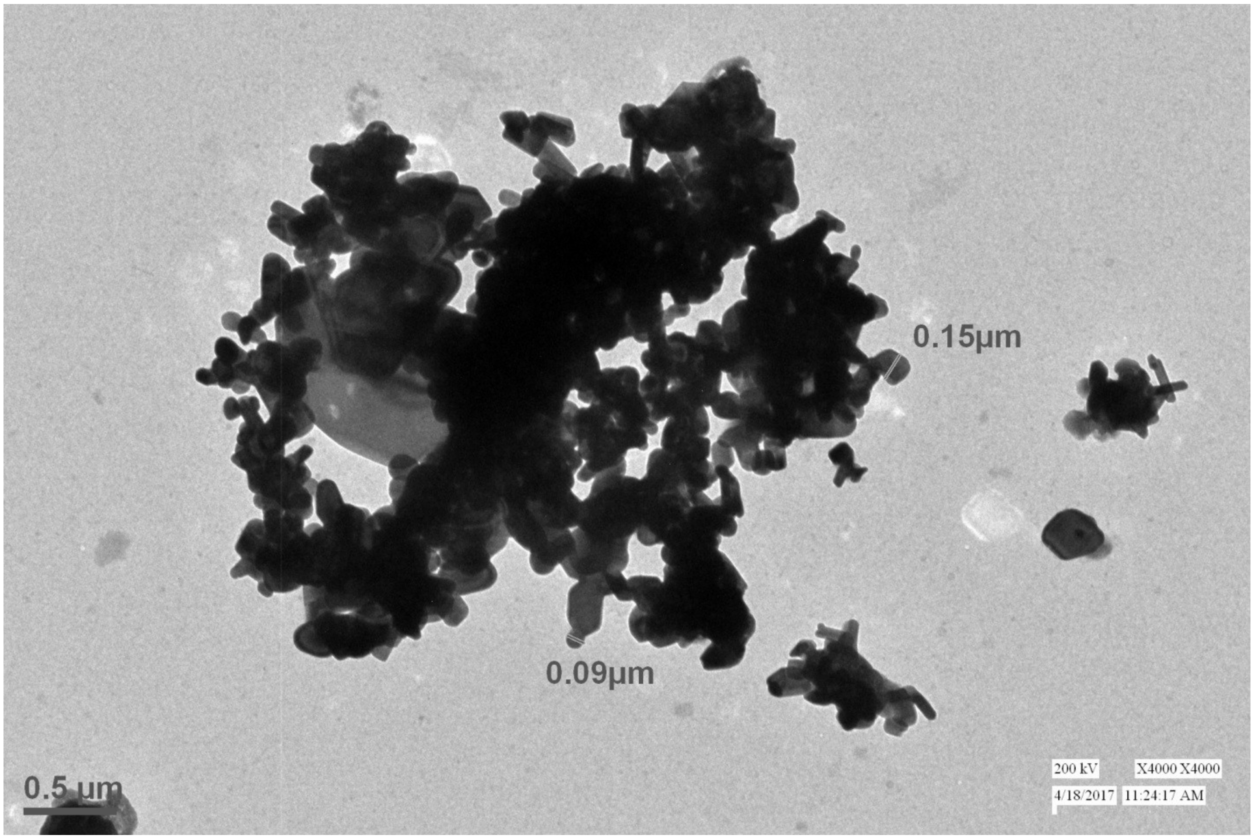
0.15μm
0.09μm
0.5 μm
200 kV
X4000 X4000
4/18/2017
11:24:17 AM

(b)

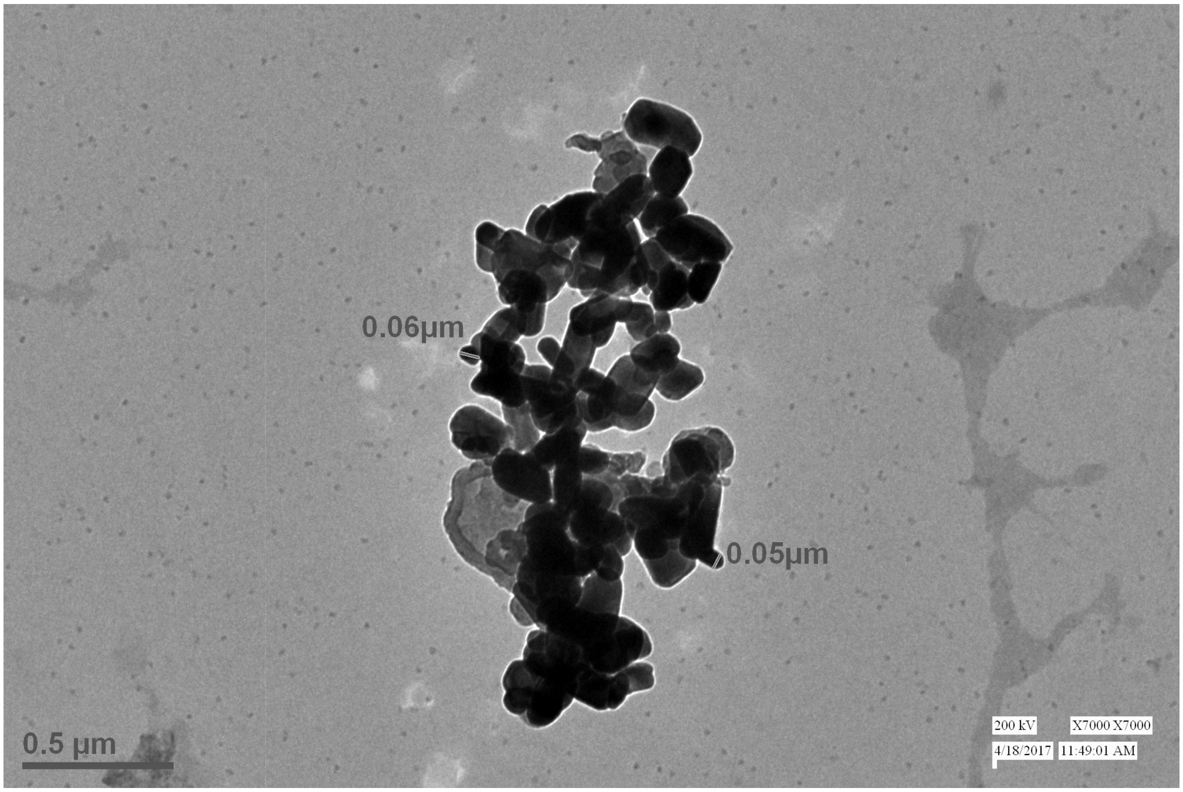
0.06µm
0.05µm
0.5 µm
200 kV
X7000 X7000
4/18/2017
11:49:01 AM

(c)

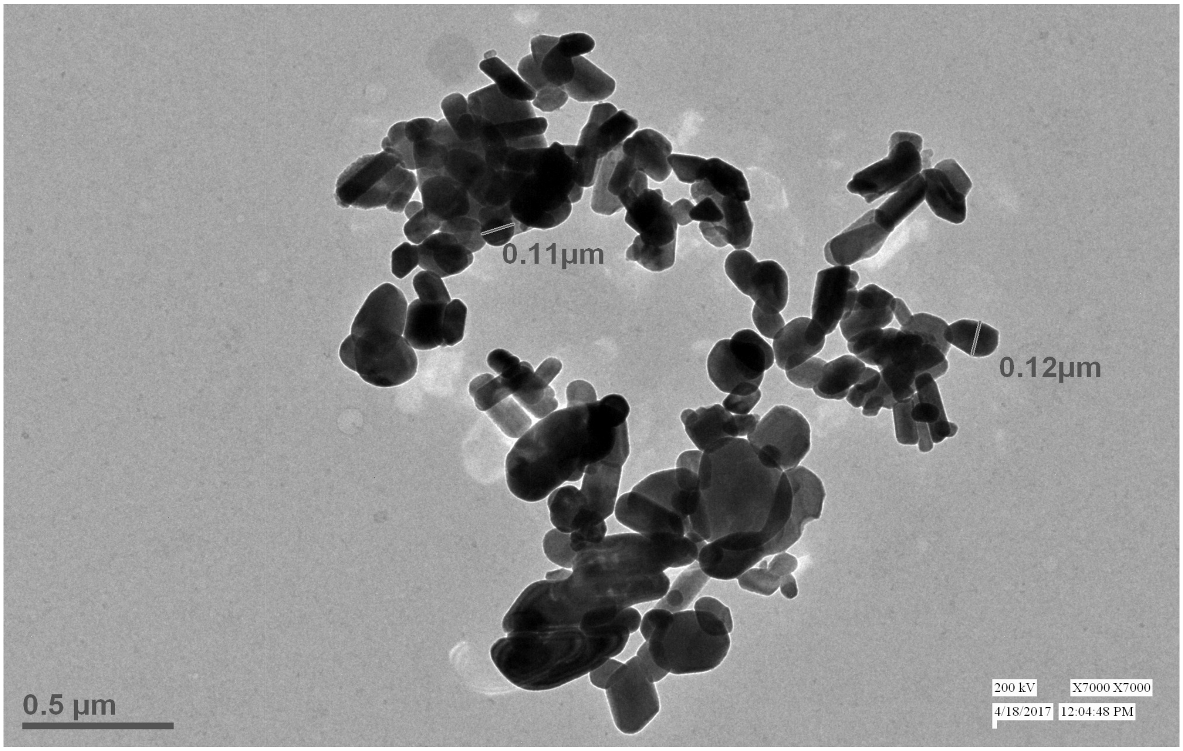
0.11µm
0.12µm
0.5 µm
200 kV
X7000 X7000
4/18/2017
12:04:48 PM

(d)

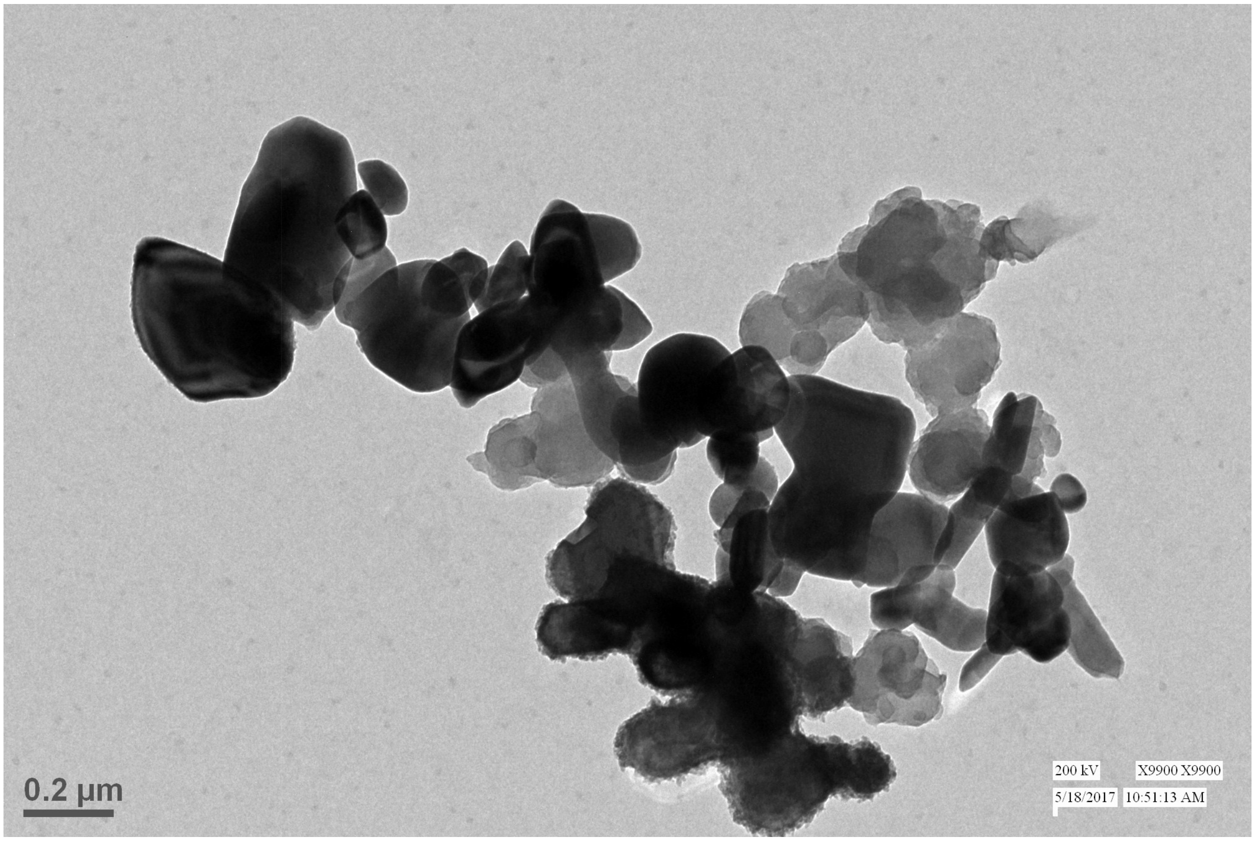

(e)

Figure S3 HRTEM of (a) Ppy (b) $AgFeO_2$, (c) 20%-Ppy/$AgFeO_2$ (d) 60%-Ppy/$AgFeO_2$, (e) 80%-Ppy/$AgFeO_2$

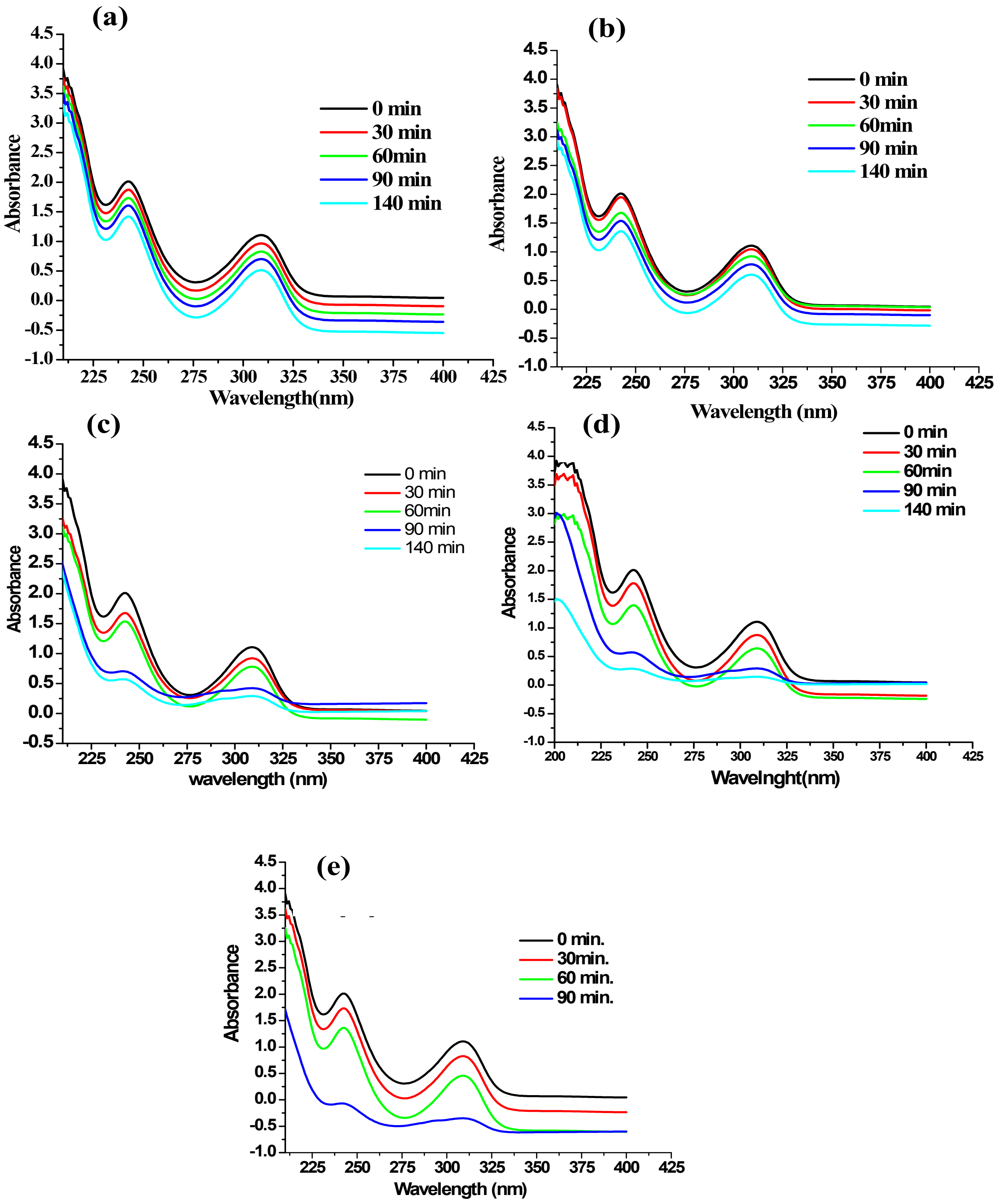

Figure S4 UV spectra of degradation of 2,4,6-TCP using (a) Ppy, (b)$AgFeO_2$, (c) 20%-Ppy/$AgFeO_2$ (d) 60%-Ppy/$AgFeO_2$ and (e) 80%-Ppy/$AgFeO_2$
